# A Simple Model Setup Using Spray‐Drying Principles and Fluorescent Silica Nanoparticles to Evaluate the Efficiency of Facemask Materials in Terms of Virus Particle Retention

**DOI:** 10.1002/admt.202100235

**Published:** 2021-05-04

**Authors:** Maximilian Oppmann, Sarah Wenderoth, Thomas Ballweg, Benedikt Schug, Karl Mandel

**Affiliations:** ^1^ Fraunhofer‐Institute for Silicate Research ISC Neunerplatz 2 D97082 Würzburg Germany; ^2^ Chair of Chemical Technology of Materials Synthesis Julius‐Maximilians‐University Würzburg, Röntgenring 11 D97070 Würzburg Germany; ^3^ Departement of Chemistry and Pharmacy Inorganic Chemistry Friedrich‐Alexander University Erlangen‐Nürnberg (FAU) Egerlandstrasse 1 D91058 Erlangen Germany

**Keywords:** aerosol, Corona, COVID‐19, facemask, FFP2, mask test, virus barrier

## Abstract

Herein, a simple model setup is presented to spray fine liquid droplets containing nanoparticles in an air stream transporting this toward a filter material. The nanoparticles are made of silica and tagged with a fluorescent dye in order to render the trace of the particles easily visible. The silica nanoparticles, in a first approximation, mimic virus (severe acute respiratory syndrome coronavirus 2) particles. The setup is used to evaluate different tissues, nowadays, in times of the coronavirus pandemic, commonly used as facemasks, with regard to their particle retention capability. The setup enables adjusting different “breathing scenarios” by adjusting the gas flow speed and, thereby, to compare the filter performance for these scenarios. The effective penetration of particles can be monitored via fluorescence intensity measurements and is visualized via scanning electron micrographs and photographs under UV light. Ultimately, a strong increase of particle penetration in various mask materials as function of flow speed of the droplets is observed and an ultimate retention is only observed for FFP3 and FFP2 masks.

## Graphical abstract

Herein, an approach to easily test the retention capability of different mask materials toward aerosol droplets, filled with nanoparticles, mimicking virus particles, is presented. This is done using a modified spray‐drying setup that can create a stream of aerosol droplets filled with fluorescent silica nanoparticles hitting toward a target tissue. Penetrated fluorescent particles are collected and visualized downstream.

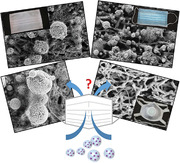

## 1 Introduction

The outbreak and worldwide spread of severe acute respiratory syndrome coronavirus 2 (SARS‐CoV‐2) in 2020 revealed the vulnerability of our human society to new types of respiratory viral infections. The course of the pandemic, especially the great speed of virus mutations and the resulting time pressure on vaccine and drug developers, shows that the strategy of containing and slowing down the spread is an indispensable part of strategies to fight virus pandemics now and in the future.^[^ ^1^ ^]^

The measures to limit or slow down the propagation consist in interrupting the transmission paths by physical distancing, reduction of social contacts, hand hygiene, and wearing masks.^[^ ^2^ ^]^ In this respect, masks play a central role as they act as a double barrier between the potential emitter and potential receiver when used consistently.

Respiratory protection has become increasingly important not only since the outbreak of the Covid 19 pandemic. Aerosol‐transmitted diseases such as tuberculosis and measles as well as influenza, SARS, and Ebola have raised awareness and alerted the health authorities. Before the SARS‐CoV‐2 epidemic, two types of masks were predominant and represented the standard: the *surgical masks (medical masks)* and the *filtering facepiece respirators* (FFR). The original function of surgical masks is to protect patients during an operation from infections by the transmission of droplets, germs, or viruses by the operating staff.^[^ ^3^ ^]^ FFRs on the other hand were designed to exclusively protect the wearer.^[^ ^4^ ^]^

In 2017 Rengansamy et al. compared different test methods to evaluate face masks and respirators, in order to facilitate a proper mask/respirator selection.^[^ ^5^ ^]^

The methods under investigations, which are explained for convenience in the following in more detail, were the so‐called NIOSH (US National Institute for Occupational Safety and Health) NaCl test, the Particulate Filtration Efficiency (PFE) test, the Bacterial Filtration Efficiency (BFE) test, and a Viral Filtration Efficiency (VFE) test.^[^ ^6^, ^7^, ^8^ ^]^

The *NIOSH* NaCl test is the N95 respirator test (42 code of federal regulations (CFR) part 84). The test is based on neutralized nanosized (75 nm) NaCl particles generated from a 2% NaCl solution. The filter efficiency is measured using an automated filter tester (TSI 8130), while the particle concentrations are measured by a light scattering photometer.^[^ ^8^ ^]^

The *PFE test* is based on a dry aerosol generation of 0.1 µm polystyrene latex particles according to American Society for Testing and Materials (ASTM) 2299 standard. The filtration efficiency is calculated by comparing the number concentrations between the upstream and downstream of the test filter measured by an optical particle counter.^[^ ^6^ ^]^

*The BFE test* determines the mask filtration efficiency based on ASTM F2101 standard. A suspension of *Staphylococcus aureus* is aerosolized using a nebulizer. The bacterial droplets, which have a mean particle size of 3.0 ± 0.3 µm, are delivered to the filtration media at a constant flow rate. The BFE is calculated by comparing the samples collected with and without the test filter media, respectively.^[^ ^7^ ^]^

The *VFE* is not recognized as a standard test method. It was adapted from the ASTM F2101 method by the Nelson Laboratories (https://www.nelsonlabs.com).^[^ ^9^ ^]^ The VFE test follows the same procedure as it is the case for BFE, with the exception that the organism under investigation is a virus (bacteriophage phiX174) with *Escherichia coli* bacteria as the host.^[^ ^7^ ^]^

Based on these tests, different mask types are routinely tested differently and judged in terms of their quality. According to the European Norm EN 149, so‐called filtering face piece (FFP2) masks (among the three available classes FFP1–3 with increasing filtering capacity) must filter at least 94% NaCl particles.^[^ ^4^ ^]^ According to the NIOSH 42 Part 84, the US equivalent N95 facepiece respirator must retain at least 95% NaCl nanoparticles.^[^ ^8^ ^]^

Comparing standard FFR test results with test results obtained for surgical masks is difficult as for instance there is a crucial difference in the way filtration is routinely evaluated according to the standard test protocols: surgical mask filtration tests are performed on a cross‐section of the masks, whereas FFRs are tested for filtration across the entire surface.^[^ ^2^, ^4^, ^8^ ^]^ Companies that produce these masks have to submit these complex and comprehensive test protocols using particle generators and analyzing systems before they are allowed to bring the masks onto the market. The technological effort to do so is enormous. The device characteristics of test aerosol generators are described, e.g., by the German guideline VDI 3491 part 1, while ISO 21501‐1 describes the requirements for optical aerosol spectrometers.^[^ ^10^ ^]^

In the mention comparative study by Rengansamy et al., they tested six types of N95 FFR as well as two surgical N95 models and three normal surgical mask models in direct comparison with the different tests available.^[^ ^5^ ^]^ The most important findings were the following:

The NIOSH NaCl test is the most conservative and sensitive test with the highest detection accuracy. Only the NIOSH NaCl test shows the limits of the normal surgical masks, insofar as it measures filter efficiencies in the range of ≈54–89%, while the other methods (also particulate filtration efficiency and the viral filtration efficiency test) obtain efficiencies above near to 100% for all surgical masks, which seems unrealistic with regard to the assessment of the current virus situation. Beyond this, the test results are difficult to compare because sometimes the entire mask including the seal leakage, sometimes only a material sample was measured. “Furthermore, PFE, BFE, and VFE suffer from the lack of precision and lack of well‐defined testing protocols.”^[^ ^5^ ^]^

In response to the SARS‐CoV‐2 outbreak and the resulting temporary shortage of masks, the use of cloth and fabric masks, many of them homemade, became commonplace and has remained so to this day. However, there is only very limited data available with regard to the filtration efficiency of common cloth materials used in such masks and unclear how these could or should be evaluated via a standard procedure.^[^ ^11^ ^]^ The most systematic study on this issue was performed by Rengasamy et al. in 2010,^[^ ^12^ ^]^ who investigated the filtration performance of cloth masks and common fabric materials when exposed to 20–1000 nm sized NaCl particles. The authors found a wide variation in penetration values of aerosol particles in the 20–1000 nm range (40–97%). The penetration levels obtained for fabric materials against aerosols were much higher than for control N95 respirator filter media but, on the other hand, they were in the range that was found as well for some surgical masks.

It is known from investigations on the former Middle‐East Respiratory Syndrome‐associated coronavirus Mers‐CoV epidemic that infected persons can produce infectious droplets of varying sizes by breathing, coughing, or sneezing.^[^ ^13^ ^]^ While large droplets quickly “sediment” to the ground, small droplets can dehydrate and remain “hovering” in air, where they behave like an aerosol. Thus, transmission can take place by both, large droplets with a short‐range and small droplet nuclei, which are airborne.^[^ ^13^ ^]^

First important studies published in the context of SARS‐CoV‐2 dealt with the visualizing of speech‐generated oral fluid droplets with laser light scattering.^[^ ^14^ ^]^ In the course of the coronavirus pandemic, the comparative studies on *aerosol filtration efficiency of common fabrics* were also taken up by Konda et al.^[^ ^15^ ^]^ Similar to Rengasamy et al., they applied NaCl aerosols following the NIOSH 42 CFR Part 84 test protocol and test equipment.^[^ ^12^ ^]^

Filtration efficiencies were measured for various commonly available fabrics that are nowadays used as masks when looking on the streets. Retention of particles in the size range of ≈10 nm to ≈6 µm was investigated. It was found that cotton, natural silk, and chiffon could provide good protection, typically above 50%, in the entire 10 nm to 6.0 µm range. Silk and chiffon were found to be particularly effective in the regime < 100 nm, likely due to electrostatic effects that result in charge transfer with nanoscale aerosol particles. The harmful effect of leakages around the masks were pointed out, which can reduce efficiencies by 50% or more.^[^ ^15^ ^]^

The dilemma is that the actual effectiveness of a mask type cannot yet be comprehensively quantified, since the masks would have to be assessed in their dual function of stopping the spreading at the source, while protecting the healthy counterpart during inhalation. The overall protective function results from this interaction. In fact, two different types of masks are considered and characterized according to different test protocols. In everyday practice, however, it is not the case that the infectious person wears the surgical mask (preferably against droplets) and the healthy person wears the barrier mask of the FFP2 or FFP3 type. Therefore, the previous certified test methods also have weaknesses with regard to their role in the infectious process.

To overcome this issue, we propose an alternative method herein. Our method is to a certain extend best comparable to the PFE test. However, while this method uses dry aerosols of latex particles, we take water‐or also saliva‐simulating‐suspensions, which are more in line with virus propagation. Virus may spread not as single isolated particles, which is only the lowest size limit, but in a broad distribution of droplets or dry aggregates depending on the conditions and the situation (emitter or receiver). Other advantages of our method are the high adaptability of size and surface of the particles and the possibility, not to count the particles on their way, but to localize them at their final places, e.g., on their diffusion path through the masks etc. Our test in the current version is suited for efficiency comparison between face mask materials, but not a comprehensive characterization of the entire mask, since it does not assess the overall effectiveness of the mask in preventing the inward leakage of particles.

### 1.1 Our Approach in Detail

Our concern was to develop the basics for a more realistic virus model, which in particular allows statements to be made about the actual spreading of viruses to better understand and control future pandemic events. A method that has a greater potential for assessing especially containment concepts and measurements like masks than the state of the art allows. The idea was to synthesize harmless, respiratory non‐infectious SARS‐CoV‐2 viral analogues, label and disperse them in water or saliva‐like liquid, generate aerosols, and track their actual whereabouts, rather than counting dry particles as is the case in the standard processes.

By spraying aqueous suspensions under controlled conditions, different transmission scenarios can be simulated such as breathing in a situation of being at rest (scenario A “at rest”), and being in motion, i.e., breathing during a moderate physical activity (such as climbing stairs) (scenario B “in motion”). The transmission/transfer by droplet infection or aerosol formation can be safely studied and analyzed.

In particular, the retention capacity of different filter materials, masks, and protective devices can be analyzed and compared. The virus duplicates provided with fluorescent markers can be tracked and their final whereabouts and fate can be traced. While the technological effort was deliberately kept small for this proof of concept, it is considered as a supplement to the existing methods.

With the suspension‐based system, we achieve a more accurate and more realistic picture of what is happening than with a solution‐based system, in which the substance to be analyzed is present as a solution or solid, depending on the external conditions. In particular, it is possible, in contrast to the FFP2 test protocol, to also study the effect of aqueous aerosols and thus to realistically evaluate their retention capacity at the emitter side. Especially, we see advantages as we are able to more realistically simulate their interaction with mask materials (see disturbing effects in other approaches such as electrostatic interaction of salt particles in^[^ ^15^ ^]^). The method accounts for wet masks that get humidified by breathing air, and it can reveal surface contamination or diffusion by capillary forces through the filter materials, while the virus analogues retain their primary particulate property in every situation. In particular, aerosol formation and the associated virus concentration can be more realistically reproduced with the help of using suspensions rather than with the concentration of a salt solution.

Coronaviruses are enveloped RNA viruses and form virions with a diameter of 80–140 nm.^[^ ^16^ ^]^ They have a single‐stranded RNA genome of positive polarity. The analogues consist of surface‐modified spherical amorphous silica nanoparticles (Stöber particles), which can perfectly adapted in size and shape to the infectious originals/viruses. A striking feature of the silica nanoparticles is the adjustability of the surface properties. By means of silanization the polarity can be adapted as needed, also positive for example by for example by silanization with an aminosilan.^[^ ^17^ ^]^

Once the method has been established, which is shown in this current proof of principle study, it can easily be upgraded by adding state of the art aerosol generators and spectrometers in order to better control and study droplet size related effects.

## 2 Results and Discussion

**Figure 1:**
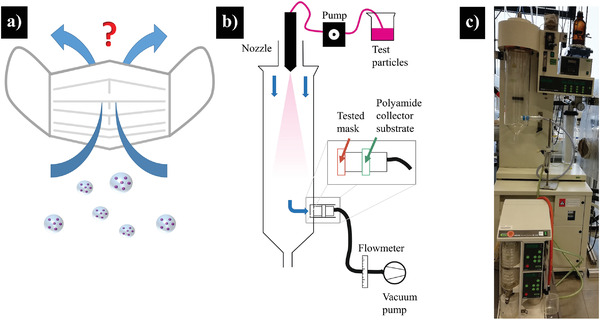
a) A facemask should prevent liquid aerosol droplets, filled with small (virus) particles ejected from mouth and nose during human breathing, from penetrating the mask tissue. b,c) Simulated process of breathing particle filled droplets via a modified spray‐drying setup (b) schematic drawing and c) photograph of the setup). With the setup, model particles (fluorescent silica nanoparticles) from a droplet spray that penetrated the mask material of interest can be found on the downstream installed collector substrate which can be analyzed afterward ex situ.

The modeling of breathing into a face mask with an artificial setup, in order to guarantee defined and reproducible measurements, comes with three challenges: first, fine droplets with solid particles inside need to be generated; second, an appropriate gas stream should take up these particles and guide them toward a tissue representing the mask material; and third, the filtered but also the penetrated fractions of particles have to be collected (**Figure** **1**, scheme a). To mimic this, a setup was designed as depicted in the scheme in Figure 1b and shown in the photograph of Figure 1c. It works as follows:

The aqueous silica model particle dispersion is delivered to the spray nozzle by a peristaltic pump and atomized by a nitrogen stream. This creates an aerosol of particle‐laden water droplets in the spray tower. The test membrane is clamped in front of the collector substrate in the laterally positioned membrane adapter. With the help of the downstream vacuum pump, the aerosol of silicate droplets is drawn through the membrane. The volume flow can be adjusted by an intermediate flow meter.

**Figure 2:**
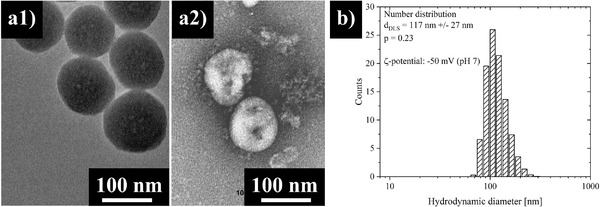
a) Electron micrographs of a1) silica nanoparticles with a mean particle diameter of 109 ± 11 nm (*p* = 0.10) and a2) real SARS‐COV‐2 particles. b) DLS measurement of silica particle dispersion in water at pH 7 with a number weighted size distribution of 117 ± 27 nm (*p* = 0.23) hydrodynamic diameter at a ξ‐potential of −50 mV. Panel (a2) reproduced with permission.^[^ ^18^ ^]^ Copyright 2020, Massachusetts Medical Society.

The droplets created in the spray‐nozzle are taken from a feed containing 109 ± 11 nm (*p* = 0.1) sized spherical, dye‐loaded Stöber silica nanoparticles (**Figure** **2**a) in deionized water (pH 7). With their size and a zeta potential of −50 mV at pH 7, the particles, in a first approximation, mimic coronavirus SARS‐CoV‐2 particles which come with a typical size of 60–140 nm.^[^ ^18^ ^]^ The nanodispersion is stable in water (size distribution measured by dynamic light scattering (DLS), Figure 2b), thus it is ensured that no larger, undefined agglomerates are present and potential agglomeration only takes place after droplets land on the filter (mask tissue). Thus, the filter/mask tissue can be tested with regard to preventing primary particle sizes (in liquid) from penetration. It should be noted that the real situation of coronavirus SARS‐CoV‐2 particles in droplets of saliva is more complex. However, the model system fulfills the crucial points of proper particle dispersion and can, therefore, be used as a first, valid approximation for the “virus in saliva” system—with the great advantage of being applicable in any lab at no risk, as amorphous silica is neither chemically nor biologically toxic and thus does not demand any particular measures of precaution.

In order to probe the model setup, it was first run with no filter/mask tissue (1), and the particle flow was collected on a polyamide collector substrate downstream of the setup (“extreme case I”: 100% particle transmission to the collector substrate). Then, the same experiment was repeated with the polyamide membrane material installed (“extreme case II”: 0% particle transmission to the collector substrate). Furthermore, a thin polyester tissue (“cloth”) mask and the well‐known standard surgical mask (white and light blue mask, used all over the world) and FFP2 and FFP3 masks were investigated, along with some other systems. For the investigations, two scenarios were analyzed:

• Scenario A “at rest”: relaxed breathing such as in a situation of being at rest, corresponding to a flow rate of the particle containing liquid droplets of 5.5 cm s^−1^.^[^ ^12^ ^]^
• Scenario B “in motion”: being in motion, i.e., breathing during a moderate physical activity (such as climbing stairs), corresponding to a flow rate of the particle containing liquid droplets of 16.5 cm s^−1^.^[^ ^12^ ^]^

**Figure 3:**
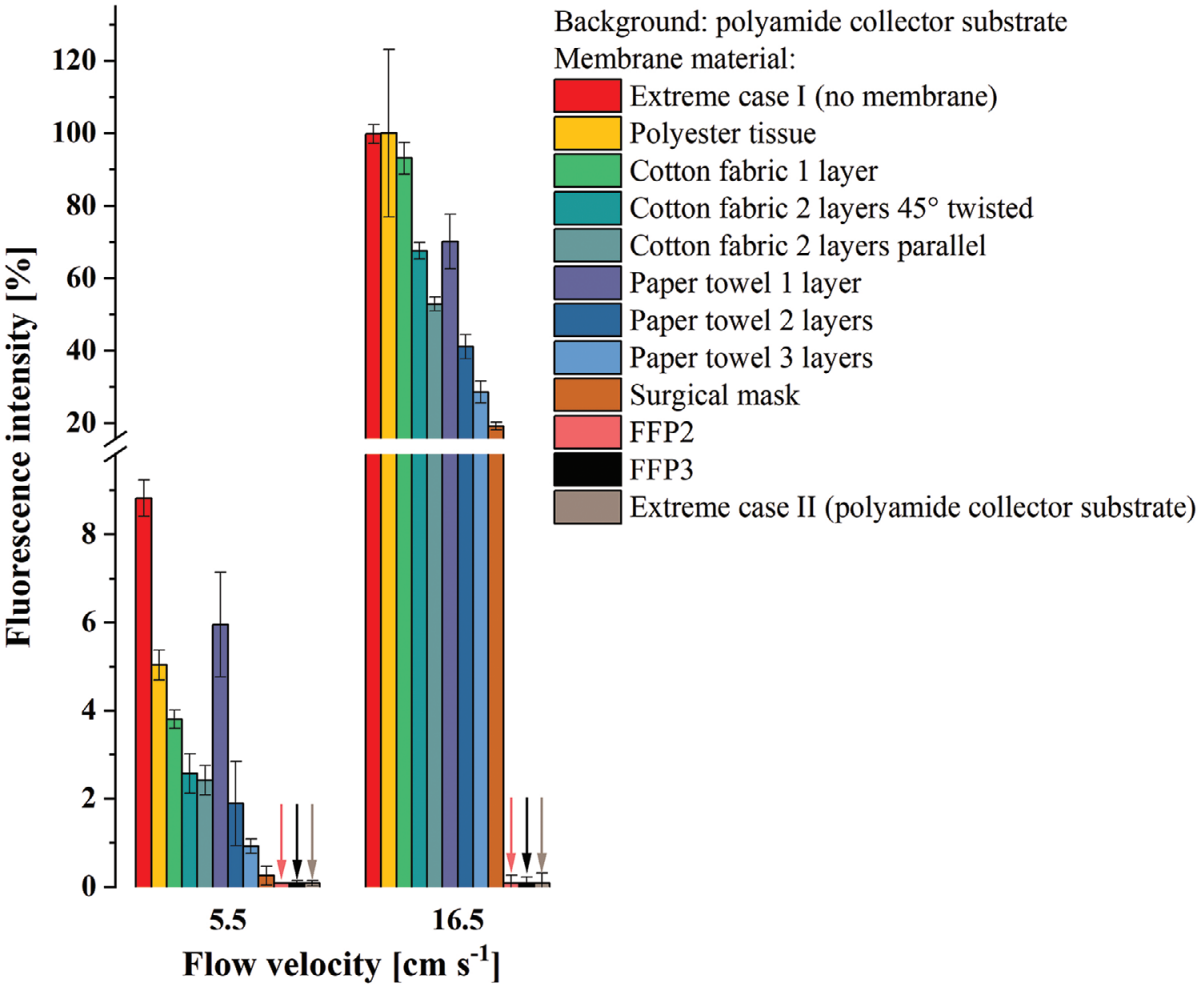
Fluorescence intensity measured on the downstream collector substrate, originating from particles that penetrated different mask materials, for two scenarios: Scenario A “at rest,” corresponding to relaxed breathing (droplet speed: 5.5 cm s^−1^) and scenario B “in motion,” corresponding to breathing, e.g., while climbing stairs (droplet speed: 16.5 cm s^−1^).

The fluorescence, measured on the collector substrate, to which each nanoparticle that penetrated the barrier contributes to, is depicted in **Figure** **3**. This figure displays the intensity values taken at the maximum fluorescence intensity at an optical wavelength of 579 nm.

An increase in fluorescence intensity can be attributed to an increased amount of nanoparticles.

Thus, fluorescence allows for a relative classification of the masks in rank order according to their retention capacity. However, an absolute assessment of the retention capacity should be treated with caution, since a linear correlation between fluorescence signal and the particle number may be disturbed by factors such as changed flow profile resulting in changed upstream losses, distribution inhomogeneities of the particles on the adsorber surface, multiple occupancy, or shielding effects of the adsorber fabric of the particles immersed/infiltrated in the adsorber material.

The results give hints how mask tissues reduce the amount of particle penetration. When looking at the diagrams in Figure 3, the first thing that stands out however is the huge difference between the scenario “in motion” compared to the scenario “at rest.” The fluorescence intensity (corresponding in a first order approximation to the number of penetrated particles per unit time) increases up to an order of magnitude. This means that beyond the increased flow rate (factor 3), the increased kinetic energy of the droplets/particles significantly promotes the penetration through the different fabrics disproportionally.

Looking at the mask types, we also see large differences. With the two boundary conditions “extreme case I” and “extreme case II,” respectively, it is possible to get an order of magnitude estimate and relative comparison of the efficiencies of the different mask materials in terms of preventing particle penetration. The poorest performance show masks made of polyester tissue, which is even without effect at higher flow rates. This is entirely plausible, as it is a hydrophobic fabric with the lowest affinity for a water‐based aerosol. The highest retention capability on the other hand has the FFP3 and the FFP2 mask followed by the surgical mask, which is not surprising, too, since all three are “professional” masks in the end. The cotton and paper based masks occupy the intermediate positions with increasing retention capability when the number of layers are increased. The retention capability of these cotton‐ or paper based homemade masks in in the range of 50% up to 80%. They provide a detectable but after all insufficient protection effect.

**Figure 4:**
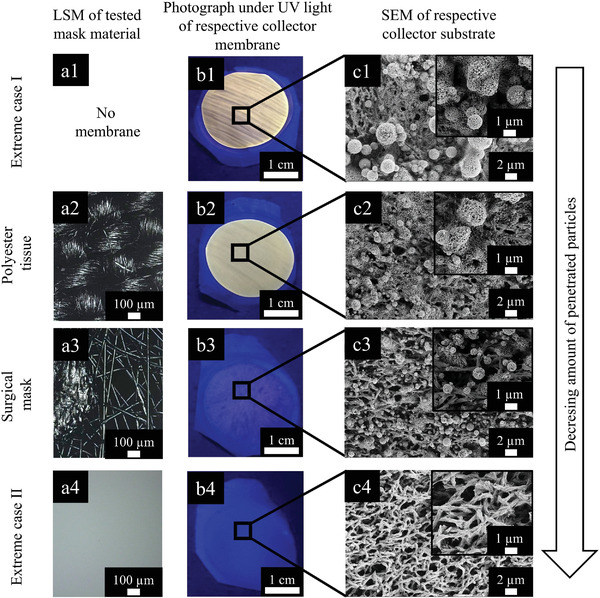
a) LSM images of the fabric structure of the mask materials: a1) no mask at all, a2) polyester tissue/“cloth” mask, a3) surgical mask, and a4) “extreme case II” (polyamide filter membrane). b) Photographs of the collector substrates installed downstream under UV‐light for the same cases as in (a). c) SEM images of the collector substrates depicting penetrated particles (insets: higher magnification). It should be noted that the fluorescence of the particles appears orange, the polyamide collector substrate itself appears in blue (images in (b)). (b) and (c) are results obtained from the simulated scenario B (“in motion”).

**Figure** **4** provides laser scanning microscope (LSM) images of the fabric structure of the three mask cases in focus (extreme case II, polyester tissue/“cloth” mask and surgical mask) (Figure 4a), photographs (under UV light) (Figure 4b) and scanning electron microscopy (SEM) images (Figure 4c) of the collector substrates installed downstream for the simulated scenario B (breathing in motion). The LSM images (Figure 4a) exemplarily reveal how different the fabric structure can look like, which makes it understandable why significant differences between the materials can be expected with respect to particle prevention. The SEM images (Figure 4c) give a good idea of the particle load transmitted and the photographs of the downstream collector (Figure 4b) remarkably give a visual impression of the high amount of solid particles (here fluorescing in orange color) that apparently were capable of penetrating the mask tissues. The combination of SEM investigations with the fluorescence studies together are capable of providing a quite good overall picture of the real situation prevailing with the tissue of interest.

**Figure 5:**
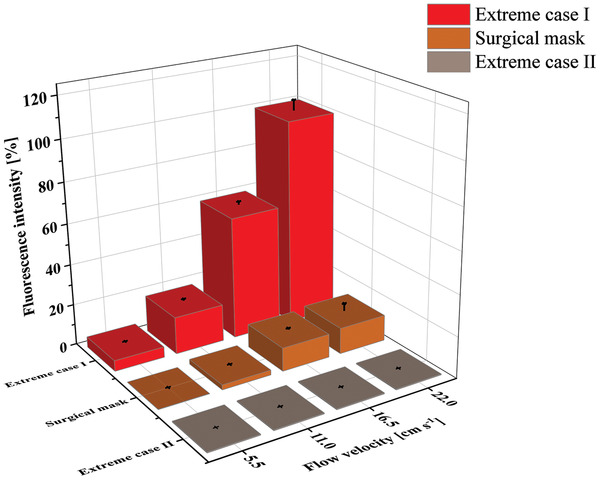
Fluorescence intensity, corresponding to the particle amount deposited on the collector substrate downstream as function of the flow velocity of the gas, carrying the wet droplets with the fluorescent nanoparticles for different “upstream cases,” namely “extreme case I” (no membrane), “surgical mask,” and “extreme case II” (polyamide collector substrate).

With the established setup, different flow speeds are easily adjustable, i.e., in order to simulate further “breathing types” such as coughing. Exemplarily, this is depicted in **Figure** **5**. It is clearly visible that fluorescence intensity, which originates from the amount of particles that landed on the collector substrate, increases with increased flow speed if no barrier is installed upstream (extreme case I). In case of a dense barrier such as the polyamide membrane (extreme case II), no fluorescence, i.e., no particle penetration is observed, no matter how much the flow speed is increased. For the sample case “surgical mask” it can be seen that the fluorescence intensity at first increases steadily for increasing flow speeds but reaches a certain “saturation value” for high flow speeds. An increase of penetrated particles in this case only up to a certain flow speed can be explained by remembering that the setup is designed in a way that solid nanoparticles in liquid droplets are sprayed on the mask tissue. At high flow speeds, it can be assumed that more moisture reaches the mask, ultimately causing a wetting of the mask, i.e., the humidity of the mask tissue increases. This potentially causes more nanoparticles to stick to this humid/wet surface, hindering penetration to a greater extent, thus ultimately causing an overall “saturation” with regard to penetration.

**Figure 6:**
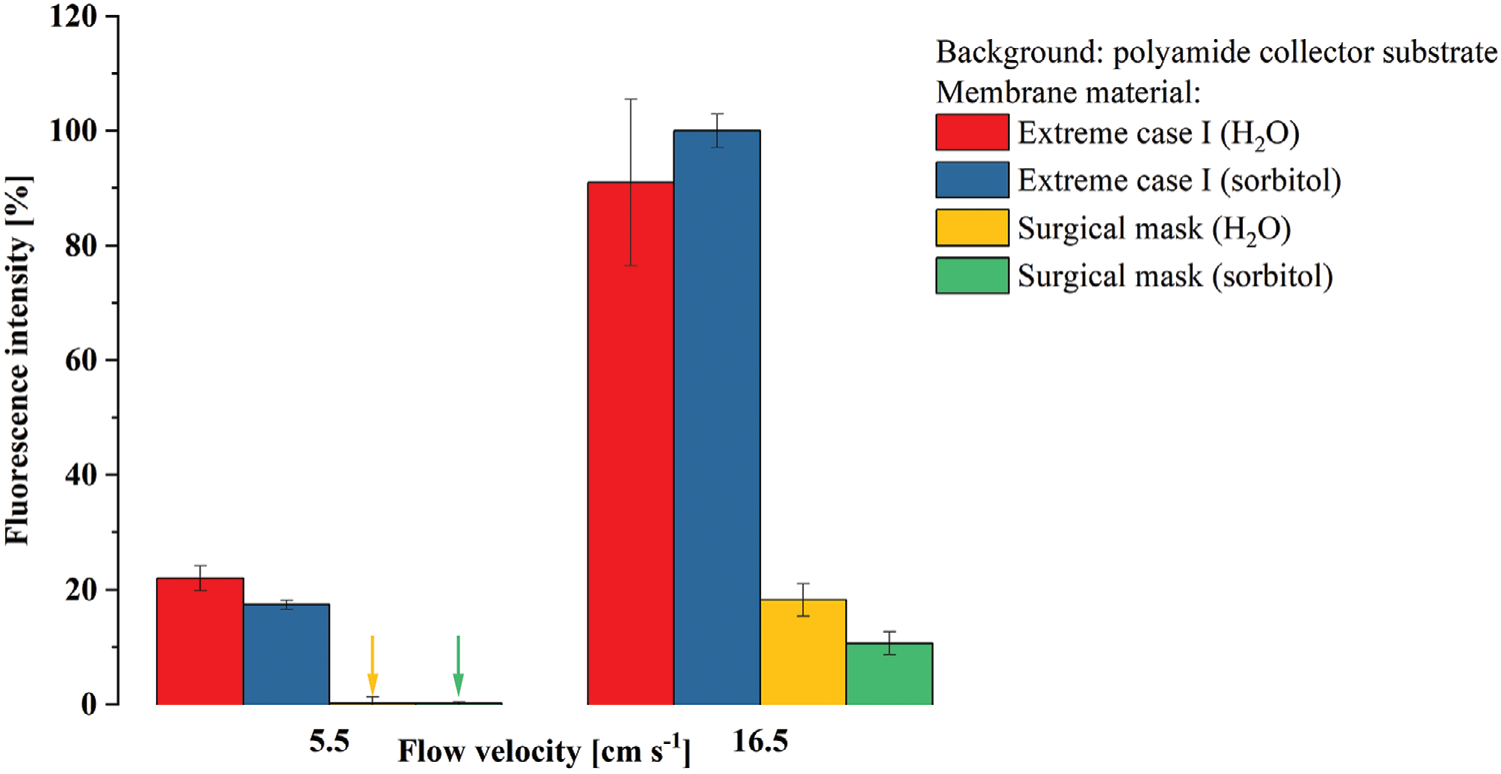
Fluorescence intensity measured on the downstream collector substrate, originating from particles dispersed in water and sorbitol solution (mimicking saliva) that penetrated a surgical mask for two scenarios: Scenario A “at rest,” corresponding to relaxed breathing (droplet speed: 5.5 cm s^−1^) and scenario B “in motion,” corresponding to breathing, e.g., while climbing stairs (droplet speed: 16.5 cm s^−1^).

As the former experiments are prepared in water, we have also done the comparison between water and an aqueous sorbitol solution (**Figure** **6**). Sorbitol is the main and dominating component in artificial salvia tests. Besides this, dissolved salts are ingredients which are neglected herein as these would cause uncontrolled agglomeration of silica nanoparticles. This, however, would not be a realistic scenario for virus particles in saliva as these are not prone to agglomeration in a saliva environment due to their protein corona.

In the “saliva” experiment, we compared the “extreme case I” and the “surgical mask” at two different flow velocities (5.5 and 16.5 cm s^−1^). No significant differences in the behavior between water and sorbitol solution as solvent could be observed. We therefore conclude that the specific liquid composition is not the crucial factor in the setup and that water is suitable as test medium to obtain meaningful results.

## 3 Conclusion

We presented a simple alternative model setup to simulate wet, nanoparticle‐containing droplet exhaust, and the penetration of the particle‐containing aerosols through different facemasks materials. This approach is expected to provide more realistic information on the fate of the virus‐analogues respectively the suitability and filtering capacity of the broad range of different materials used for facemasks today.

The test results indicate that mask tissues can reduce particle transmittance. However, they also indicate that there are differences in mask tissues and that it clearly matters which tissue is used and not all masks can be considered to perform equally well. Further, the increase in flow speed, i.e., corresponding to a more excited breathing, drastically increases the amount of expelled particles and also particles that penetrate a mask tissue. Still, any mask material reduces the amount of penetrated particles but some mask materials are more suitable. In our study, results suggest that among all tested mask materials ultimately, only highly dense barriers such as in FFP3 and FFP2 masks prevent silica nanoparticle penetration.

Of course, in detail, a differentiation has to be made between a barrier meant for preventing particles to leave “an emitter” (= the particles from the own breath of the wearer of the mask) and a barrier meant for protecting “a receiver” (= the particles from someone else's breath hitting the mask of the wearer). The reason is that the distance of droplet‐emitting‐source to barrier is different and with this, droplet speed, moisture level, and size distribution. With this respect, the particular strength of our suspension‐based system is in particularly seen with respect to studying the case of droplet emission (= emitter‐side) in comparison to the solution‐based state of the art system used to date.

## 4 Experimental Section

##### Materials

All chemicals were purchased from Sigma‐Aldrich (Germany) except for the aqueous ammonia solution, which was purchased from VWR (Germany). All chemicals were used as received without further purification. All experiments were carried out at room temperature.

##### Fluorescent Stöber Silica Nanoparticle Synthesis as a Virus Analogue

The synthesis was based on the well‐known protocol of Stöber et al.^[^ ^19^ ^]^ For this purpose, 600 mL ethanol were mixed with 26.2 g 25% aqueous ammonia solution and 13.1 g deionized water. After 5 min stirring, 30.1 g (0.144 mol) tetraethyl orthosilicate was rapidly added and stirred for 30 min before 600 µL of a previously prepared dye aliquot was added. The dispersion was stirred for further 24 h and was purified by rotary evaporation and dialysis against deionized water (4 L, water exchanges after 30, 90, 240, and 480 min) for 24 h.

The dye aliquot was prepared by dilution of 9 mg (0.017 mol) of rhodamine B isothiocyanate in 2 mL dimethyl sulfoxide and mixing with 13 µL (0.055 mmol) 3‐aminopropyltriethoxysilane.^[^ ^20^ ^]^

##### Mask Material Test Setup Parameters

In this study, mainly fabrics were tested which are relevant to the everyday life of the general population and are used for so‐called everyday masks/community masks. A polyester tissue, a cotton fabric (one and two layers) as well as a simple paper towel (one, two, and three layers), which can be used for the construction of a homemade mask, were subject to investigations. For the cotton fabric, the influence of the orientation of the fabric (parallel and 45° twisted) was also investigated for a two‐layer construction.

In addition to these everyday mask materials, the membrane material of a surgical surgical mask (= the well‐known mask with one blue and one white side), a professional FFP2 mask and a professional FFP3 mask were tested.

The materials were tested using the atomizer unit and spray tower of a BÜCHI B290 spray‐dryer (BÜCHI, Switzerland). The aqueous silica particle dispersion (solids content 1.5%) was pumped at a rate of 0.3 mL min^−1^ and atomized with a two‐fluid nozzle (two‐fluid nozzle flow: 357 L h^−1^). Thereby, aerosol droplets in the size range between 5 and 50 µm are generated.^[^ ^21^ ^]^ One experiment has a time duration of 15 min. The membrane adapter with the test membrane and the collector substrate was positioned in the lateral outlet of the spray tower. During the experiment, the relative humidity in the spray tower is about 100% at 21 °C. A vacuum pump (BÜCHI Vacuum System B‐179) with flowmeter was connected to the membrane adapter. The volume flow could be varied between 100 and 400 L h^−1^. The area of the test membranes was 5 cm^2^, resulting in the relationship between volume flow and flow velocity shown in **Table** **1**.

**Table 1:** Relationship between volume flow and flow velocity

Volume flow [L h^−1^]	Flow velocity [cm s^−1^]
100	5.5
200	11
300	16.5
400	22

The experiments in sorbitol solution were done in a sorbitol solution which corresponds to the artificial saliva neues rezeptur formularium (NRF) 7.5 composition: 4.3 g of a 70% sorbitol solution was dissolved to 100 mL water. The particle dispersion in water was centrifuged and dispersed in this sorbitol solution again.

##### Analyses

Fluorescence spectroscopy measurements were conducted by using a Jasco FP‐8600 spectrofluorometer (Jasco, Japan) with a cuvette sample holder for the particle analysis, and with a solid sample holder (FDA‐808, Jasco, Japan) for the mask tests. The excitation wavelength was set to 540 nm and the spectra were recorded between 560 and 700 nm.

The detection of the morphology and mean particle diameter of the silica particles were determined by using a JEM‐2010 from (Jeol, Japan) with an acceleration voltage of 200 kV. The particle size distribution was recorded by calculating more than 80 particles using the X‐ImageJ software.

The hydrodynamic diameter and ζ‐potential of the silica particles in dispersion were detected by using a Zetasizer Nano ZS (Malvern Instruments, United Kingdom). Measurements were performed at 25 °C and in a triplicate run.

The detection of the morphology of the different mask materials were performed by using an LSM Keyence color 3D VK‐X210 (Keyence, Germany).

The morphology of the polyamide collector substrate and the collected silica particles was studied via SEM on a Zeiss Supra 25 (Zeiss, Germany) at 1.5 keV (field emission) and a secondary electron sensitive detector.

## Conflict of Interest

The authors declare no conflict of interest.

## Data Availability

Research data are not shared.
